# Coffee Anthracnose-Associated Fungi from Wanning, Hainan: Fungicide Sensitivity and Broad-Spectrum Antagonism by Mangrove-Derived *Streptomyces* sp. CD103

**DOI:** 10.3390/jof12100712

**Published:** 2026-09-23

**Authors:** Jiajun Li, Jiayi Li, Zhicheng Zheng, Wenxin Yuan, Rongsuo Hu, Xiaoxiong Xu, Huimin Feng

**Affiliations:** 1 Key Laboratory for Tropical Crop Molecular Breeding, Hainan Tropical Ocean University, Sanya 572022, China; lijiajun19970226@163.com (J.L.); 18389556049@163.com (J.L.); 15256180631@163.com (Z.Z.); a17839425365@163.com (W.Y.); 2 Spice and Beverage Research Institute, Chinese Academy of Tropical Agricultural Sciences, Wanning 571533, China; hnhrs@126.com

**Keywords:** coffee anthracnose, *Colletotrichum*, fungicide sensitivity, mangrove-derived actinomycetes, *Streptomyces*

## Abstract

Fungi associated with coffee leaf lesions provide a diverse target panel for evaluating fungicide sensitivity and microbial antagonism. This study evaluated the antagonistic spectrum of mangrove-derived *Streptomyces* sp. CD103 and fungicide sensitivity in selected lesion-associated fungi. Of 49 isolates recovered from symptomatic *Coffea arabica* leaves in Wanning, Hainan, 39 induced necrosis following wound inoculation. These isolates were assigned to eight genera based on internal transcribed spacer (ITS) sequence comparisons and phylogenetic placement; *Colletotrichum* accounted for 64.1% of the necrosis-inducing isolates. Six fungicides were tested against seven isolates spanning four genera. Sensitivity varied among isolates and compounds; concentrations causing 50% inhibition of mycelial growth (EC_50_) for thiophanate-methyl ranged from 0.57 to 22.53 µg mL^−1^. Among 45 mangrove-derived actinomycetes screened against *Colletotrichum gloeosporioides* CCG3, CD103 showed the highest mean inhibition (99.21 ± 0.40%). In subsequent dual-culture assays, CD103 completely inhibited 19 of the 21 selected lesion-associated isolates spanning eight genera and all six reference plant pathogens. The two exceptions, a *Fusarium* isolate and an *Alternaria* isolate, showed 85.04% and 97.10% inhibition, respectively. These findings identify CD103 as a broad-spectrum in vitro antagonist against the tested fungi and warrant further evaluation of its disease-suppressive potential on coffee plants.

## 1. Introduction

Coffee anthracnose, primarily associated with *Colletotrichum* spp., is an important fungal disease in coffee-growing regions. In Hainan, eight *Colletotrichum* species have been recovered from symptomatic coffee leaves and berries, and a recent survey in China further revealed substantial diversity and variation in pathogenicity among coffee-associated *Colletotrichum* taxa [1,2]. Fungi outside *Colletotrichum*, including species of *Fusarium* and *Diaporthe*, have also been recovered from symptomatic coffee tissues [3]. This diversity provides a basis for assembling culture collections to evaluate antifungal activity across taxonomically distinct fungi associated with coffee lesions.

Species delimitation within *Colletotrichum* is complicated by the presence of numerous closely related species complexes, for which internal transcribed spacer (ITS) sequence data alone often lack sufficient resolution for species-level assignment [4]. Multilocus phylogenetic analyses of coffee-associated *Colletotrichum* have likewise demonstrated considerable diversity within these complexes [1,2,5]. For antifungal screening, genus-level assignments based on ITS sequence comparisons and phylogenetic placement provide a practical framework for organizing recovered isolates and selecting taxonomically diverse representatives for subsequent assays.

Isolate-dependent variation is particularly relevant to chemical control. *Colletotrichum* populations isolated from rubber trees in Hainan exhibited marked variation in sensitivity to carbendazim, chlorothalonil, and demethylation-inhibiting fungicides [6]. Similarly, *Colletotrichum siamense* and *Colletotrichum fructicola* isolates associated with strawberry anthracnose displayed distinct sensitivity profiles when exposed to different fungicides [7]. These findings support evaluating fungicide sensitivity across multiple isolates. Important constraints associated with chemical fungicide use include the development of resistance in pathogen populations, environmental contamination, and potential toxicity to non-target organisms [8,9,10].

Microbial antagonists offer a complementary strategy for suppressing anthracnose-associated fungi and reducing reliance on chemical fungicides. A bioformulation of *Chaetomium cupreum* demonstrated efficacy against coffee anthracnose caused by *Colletotrichum gloeosporioides* [11]. Members of the genus *Streptomyces* are particularly attractive as sources of antifungal agents because many strains produce bioactive secondary metabolites and extracellular enzymes. Notably, extracts from the mangrove-derived strain *Streptomyces* sp. HSL-9B inhibited *C. gloeosporioides* and reduced mango anthracnose in fruit assays [12]. Broad-spectrum antifungal activity was also reported for crude extracts of *Streptomyces* sp. SCA3-4 against 13 phytopathogenic fungi, including multiple *Colletotrichum* species [13]. For coffee-associated fungi, a key question is whether antagonists selected against a reference pathogen retain activity across a broader range of lesion-associated isolates.

The primary objective of this study was to identify a mangrove-derived actinomycete with broad in vitro antagonistic activity against fungi associated with coffee leaf lesions. We established a culture collection from symptomatic *Coffea arabica* leaves in Wanning, Hainan, and used wound-induced necrosis as a screening criterion for inclusion in the test panel. The retained isolates were characterized using colony traits and ITS-based phylogenetic placement at the genus level. Six fungicides were evaluated against seven selected isolates spanning four genera to characterize variation in fungicide sensitivity. In parallel, 45 mangrove-derived actinomycetes were screened against *C. gloeosporioides*, and the selected strain, *Streptomyces* sp. CD103, was subsequently evaluated against 21 lesion-associated isolates spanning eight genera and six additional reference plant pathogens to determine the breadth of its in vitro antagonistic activity.

## 2. Materials and Methods

### 2.1. Sample Collection, Microbial Strains, and Culture Media

Field sampling was conducted on 2 November 2024 at the Spice and Beverage Research Institute, Chinese Academy of Tropical Agricultural Sciences (CATAS), Wanning, Hainan Province, China (18.735195° N, 110.192177° E). Fifty leaves exhibiting anthracnose-like symptoms, characterized by brown-to-black necrotic lesions, zonation, or concentric rings, were collected from 10 *Coffea arabica* plants. Collected leaves were placed individually into sterile polyethylene bags, transported to the laboratory at 4 °C, and processed within 24 h.

Potato dextrose agar (PDA) was prepared using commercial potato dextrose medium (HB0233-4; Qingdao Hope Bio-Technology Co., Ltd., Qingdao, China) supplemented with agar powder (BS195; Beijing Labgic Technology Co., Ltd., Beijing, China) and used for fungal isolation, purification, activation, fungicide sensitivity assays, and dual-culture tests. International *Streptomyces* Project medium 2 (ISP2) agar (HB8746; Qingdao Hope Bio-Technology Co., Ltd.) was used to activate mangrove-derived actinomycetes and prepare mycelial agar blocks.

Six reference plant pathogens were included to evaluate the antagonistic breadth of the selected actinomycete. *Botrytis cinerea* HF 2008148 was obtained from the China Center for Type Culture Collection (CCTCC, Wuhan, China). *Fusarium oxysporum* Foc4, *C. gloeosporioides* CCG3, *C. musae* CM01, *Phytophthora cinnamomi* ZH01, and *P. nicotianae* Pb2202 were kindly provided by the Environment and Plant Protection Institute, CATAS (Haikou, China). Among these, *C. gloeosporioides* CCG3 served as the target strain for the primary screening of the 45 candidate actinomycetes. The 45 candidate actinomycetes had been isolated from sediments collected at 12 mangrove sites in Hainan and were maintained in our laboratory culture collection.

### 2.2. Isolation of Lesion-Associated Fungi and Assessment of Wound-Induced Necrosis

Fungi were isolated from symptomatic tissues using a tissue-segment method with minor modifications [1,2]. Five tissue pieces (approximately 5 mm × 5 mm) were excised from the margins between diseased and healthy tissues of each leaf, immersed in 75% (*v*/*v*) ethanol for 30 s followed by 2% sodium hypochlorite for 2–3 min, rinsed five times with sterile water, and plated on PDA. Plates were incubated at 28 °C, and emerging fungal colonies were transferred from the colony margins to fresh PDA three times to obtain pure cultures.

A wound-inoculation detached-leaf assay, adapted from previous coffee leaf inoculation assays [1,2], was used to evaluate necrosis induction. Healthy coffee leaves at a similar developmental stage were rinsed under running water for 20 min and then three times with sterile water. For each isolate, one detached leaf was wounded at three sites with a sterile needle, and a 5 mm mycelial plug was placed mycelium-side down on each wound; sterile PDA plugs served as negative controls. Leaves were maintained in humid chambers at 28 °C and evaluated at 7 d after inoculation. Isolates producing clear necrosis at the inoculation sites while the controls showed no expanding necrotic lesions were classified as necrosis-inducing. Fungi were re-isolated from lesion margins and compared with the inoculated cultures on the basis of colony morphology. This assay was used to select isolates for subsequent antifungal testing based on their ability to induce necrosis under the assay conditions.

### 2.3. Colony Morphology, ITS Sequencing, and Phylogenetic Analysis

Necrosis-inducing isolates were cultured on PDA at 28 °C for 7–14 d, and colony morphology and pigmentation were recorded. Genomic DNA was extracted from mycelia using a fungal genomic DNA extraction kit (WLA129a; Wanleibio, Shenyang, China) according to the manufacturer’s instructions. The ITS1–5.8S–ITS2 region was amplified using primers ITS1 (5′-TCCGTAGGTGAACCTGCGG-3′) and ITS4 (5′-TCCTCCGCTTATTGATATGC-3′) [14]. Each 50 µL PCR contained 25 µL of 2× Taq Master Mix, 2 µL of each primer, 2 µL of DNA template, and sterile water to volume. PCR amplification consisted of an initial denaturation at 94 °C for 5 min; 35 cycles of 94 °C for 30 s, 58 °C for 30 s, and 72 °C for 1 min; and a final extension at 72 °C for 7 min.

PCR products were verified by agarose-gel electrophoresis and sequenced bidirectionally by Boshang Biotechnology (Sanya, China). Forward and reverse reads were trimmed, assembled, and manually inspected. The assembled ITS sequences were queried using the NCBIBLASTn web service with the megablast option against the NCBI fungal ITS RefSeq database, with searches restricted to sequences from type material. The best-matching reference sequences were retrieved and deduplicated by accession number. Separate datasets were assembled for the 25 *Colletotrichum* isolates and the 14 isolates assigned to other genera, with each study isolate retained as an individual sequence. Sequences were aligned using MUSCLE as implemented in MEGA 11 [15,16] and trimmed at both ends. Maximum-likelihood phylogenetic analyses were performed in IQ-TREE 3.1.2 [17], with the best-fit nucleotide substitution model selected by ModelFinder according to the Bayesian information criterion (BIC) [18]. TIM+G4 was selected for the *Colletotrichum* dataset, whereas TIM2+G4 was selected for the non-*Colletotrichum* dataset. Branch support was assessed using 1000 SH-like approximate likelihood ratio test (SH-aLRT) replicates [19] and 1000 ultrafast bootstrap (UFBoot) replicates [20]. *Monilochaetes infuscans* CBS 869.96 and *Leotia lubrica* JMP0049 were used as outgroups for the *Colletotrichum* and non-*Colletotrichum* datasets, respectively, and the resulting trees were visualized in FigTree v1.4.4.

Genus-level assignments were based primarily on BLASTn similarity and ITS-based phylogenetic placement, with PDA colony morphology used as supporting phenotypic evidence. Study isolates were retained at genus level, and species names associated with reference sequences were reported only as BLAST matches. Newly generated ITS sequences were deposited in GenBank under accession numbers PZ804667–PZ804705. Genus-level assignments, study ITS accession numbers, and details of the best-matching type-material reference sequences are provided in Table S1.

### 2.4. In Vitro Fungicide Sensitivity Assays

Seven isolates (CA3, CA15, CA17, CA33, CA34, CB1, and CB5) were selected to provide genus-level diversity while including multiple *Colletotrichum* isolates with distinct ITS sequence profiles and stable growth on PDA. All seven isolates produced clear necrosis in the wound-inoculated detached-leaf assay. The panel spanned four genera and comprised four *Colletotrichum* isolates (CA3, CA15, CA17, and CB1), together with *Lasiodiplodia* sp. CA33, *Clonostachys* sp. CA34, and *Diaporthe* sp. CB5. Six fungicides representing methyl benzimidazole carbamates (MBCs), multisite protectants, and copper-based products were evaluated: carbendazim, thiophanate-methyl, mancozeb, chlorothalonil, Bordeaux mixture, and basic copper sulfate.

Commercial formulations were 50% carbendazim wettable powder (Sichuan Runer Technology Co., Ltd., Jianyang, China), 70% thiophanate-methyl wettable powder (Nippon Soda Co., Ltd., Tokyo, Japan), 80% mancozeb wettable powder (Limin Chemical Co., Ltd., Xinyi, China), 75% chlorothalonil wettable powder (Shandong Xinxing Pesticide Co., Ltd., Qingzhou, China), 80% Bordeaux mixture wettable powder (Tongzhou Zhengda Pesticide Chemical Co., Ltd., Nantong, China), and 27.12% basic copper sulfate suspension concentrate (Nufarm Australia Ltd., Laverton North, VIC, Australia). Fungicide concentrations were calculated on the basis of the active-ingredient content stated on the product labels and are reported as nominal active-ingredient concentrations. Fresh stock suspensions (20 mg mL^−1^) were prepared in sterile water and diluted as required.

Fungicide suspensions were added to sterile PDA cooled to 50–55 °C to obtain the concentration ranges shown in Table 1. Fungicide-free PDA served as the control. A 5 mm mycelial plug was placed at the center of each plate, and plates were incubated at 25 °C in darkness. Each treatment included three independent plates. When control colonies approached the plate margin, colony diameter was measured along two perpendicular axes. Mycelial growth inhibition was calculated as

$$I=\frac{D_{0}-D_{t}}{D_{0}-d}\times 100\%$$

where $D_{0}$ and $D_{t}$ are the mean colony diameters of the control and fungicide treatment, respectively, and d is the initial plug diameter.

Mean inhibition at each positive fungicide concentration was fitted using a nonlinear least-squares (NLS) Hill model

$$Y=\frac{100}{1+{\left(EC_{50}/X\right)}^{h}}$$

where Y is inhibition (%), X is fungicide concentration, EC_50_ is the concentration causing 50% inhibition of mycelial growth, and h is the Hill slope. The fungicide-free control (X = 0) was excluded from model fitting. The upper asymptote was fixed at 100%, and EC_50_ and h were constrained to positive values. Parameters were estimated by bounded nonlinear least squares in SciPy version 1.18.0 using multiple starting values; the solution with the smallest residual sum of squares was retained. Model fit was evaluated using the coefficient of determination (R^2^) and root-mean-square error on the original inhibition scale. Model-derived EC_50_ estimates above the highest tested concentration were retained and identified as extrapolated values. Because Bordeaux mixture produced inhibition far below 50% over the tested range, no EC_50_ model was fitted for this fungicide. Concentration-specific inhibition values and complete model outputs are provided in Table S2.

### 2.5. Screening of Mangrove-Derived Actinomycetes and Broad-Spectrum Antagonism of CD103

Candidate actinomycetes were activated on ISP2 agar at 28 °C for 10 d before dual-culture assays. In the four-point confrontation assay, a 5 mm × 5 mm block of the test isolate was placed at the center of a PDA plate, and four actinomycete blocks of the same size were positioned equidistantly 2.5 cm from the plate center. The culture surfaces of all inoculum blocks faced upward. Plates inoculated only with the test isolate served as controls. Each treatment included three independent plates, which were incubated at 28 °C in darkness for 5–7 d. When control colonies approached the plate margin, plates were photographed under standardized conditions, and colony areas of the test isolate were measured using ImageJ version 1.54g following Costa et al. [21]. Area-based inhibition for replicate i was calculated as

$$I_{i}=\frac{{\bar{A}}_{C}-A_{T,i}}{{\bar{A}}_{C}}\times 100\%$$

where ${\bar{A}}_{C}$ is the mean colony area of the three control replicates and $A_{T,i}$ is the colony area of treatment replicate i. Negative values were set to 0%. Complete inhibition (CI) was recorded when no measurable growth of the test isolate beyond the initial inoculum block occurred in any of the three replicates.

*C. gloeosporioides* CCG3 was used as the target strain for screening the 45 mangrove-derived actinomycetes, with 50% inhibition used as a screening reference. CD103 was selected on the basis of its highest mean inhibition and consistency among replicates. To assess the breadth of its antagonistic activity, 21 isolates were selected from the necrosis-inducing collection based on genus-level assignments and preliminary ITS sequence comparisons to maximize taxonomic coverage. The panel included 11 *Colletotrichum*, three *Diaporthe*, and two *Fusarium* isolates, and one isolate each of *Lasiodiplodia*, *Clonostachys*, *Alternaria*, *Nigrospora*, and *Collariella*. These 21 isolates and the six reference plant pathogens described in Section 2.1 were evaluated against CD103 using the same dual-culture assay.

Genomic DNA of CD103 was prepared by boiling extraction. The 16S rRNA gene was amplified using primers 27F (5′-AGAGTTTGATCCTGGCTCAG-3′) and 1492R (5′-TACGGYTACCTTGTTACGACTT-3′). PCR amplification consisted of an initial denaturation at 94 °C for 2 min; 35 cycles of 94 °C for 30 s, 58 °C for 30 s, and 72 °C for 2 min; and a final extension at 72 °C for 10 min. PCR products were verified by agarose-gel electrophoresis and sequenced by BGI Genomics Co., Ltd. (Shenzhen, China). The assembled sequence was compared with GenBank records using BLASTn and with type-strain sequences in EzBioCloud. The 16S rRNA gene sequence of CD103 was deposited in GenBank under accession number PV420955.1.

### 2.6. Statistical Analysis and Image Processing

Data are presented as the mean ± standard deviation (SD) where applicable. Replicate-level area-based inhibition values from the 45-actinomycete screening assay were analyzed by one-way analysis of variance (ANOVA) followed by Tukey’s honestly significant difference (HSD) test in IBM SPSS Statistics version 27, with *p* < 0.05 considered statistically significant. Fungicide concentration-response data were analyzed using the NLS-Hill procedure described in Section 2.4. Data visualization was performed in R version 4.6.0. Original plate and leaf images were cropped and resized uniformly, with only global brightness adjustments applied in Adobe Photoshop 2024; no experimental features were added, removed, or selectively altered. Figure panels, labels, and annotations were assembled in Adobe Illustrator 2024.

## 3. Results

### 3.1. Diversity and ITS Phylogenetic Placement of Necrosis-Inducing Fungi

Forty-nine fungal isolates were recovered from coffee anthracnose-like lesions. Of these, 39 isolates (79.6%) produced clear necrosis at wound-inoculation sites, whereas the sterile-PDA controls did not develop expanding necrotic lesions (Figure 1A; Figure S1). These isolates were therefore retained as necrosis-inducing fungi for subsequent characterization. Their colonies showed substantial variation in color, texture, pigmentation, concentric zoning, and margin morphology on PDA. Representative phenotypes and the genus-level composition of the necrosis-inducing collection are shown in Figure 1.

Based on ITS phylogenetic placement, BLASTn similarity, and supporting colony traits, the 39 necrosis-inducing isolates were assigned to eight genera (Figure 2; Table 2; Table S1). *Colletotrichum* was predominant, comprising 25 isolates (64.1%). *Diaporthe* comprised six isolates (15.4%), and *Fusarium* comprised three isolates (7.7%). *Lasiodiplodia*, *Clonostachys*, *Alternaria*, *Nigrospora*, and *Collariella* were each represented by one isolate (2.6%).

### 3.2. In Vitro Sensitivity of Seven Selected Isolates to Six Fungicides

The seven-isolate panel comprised four *Colletotrichum* isolates and one isolate each of *Lasiodiplodia*, *Clonostachys*, and *Diaporthe*. Five of the six fungicides produced concentration-dependent inhibition suitable for NLS-Hill fitting, yielding 35 EC_50_ estimates with R^2^ values ranging from 0.8608 to 0.9998 (Figure 3; Table 3; Table S2). Arithmetic mean EC_50_ values for thiophanate-methyl, mancozeb, chlorothalonil, carbendazim, and basic copper sulfate were 4.88, 33.46, 37.06, 82.37, and 458.58 µg mL^−1^, respectively.

Sensitivity varied substantially among isolates exposed to the same fungicide. For thiophanate-methyl, EC_50_ values ranged from 0.57 µg mL^−1^ for *Colletotrichum* sp. CA3 to 22.53 µg mL^−1^ for *Clonostachys* sp. CA34. Mancozeb EC_50_ values ranged from 3.68 µg mL^−1^ for *Lasiodiplodia* sp. CA33 to 68.29 µg mL^−1^ for *Colletotrichum* sp. CA17, whereas chlorothalonil values ranged from 3.94 µg mL^−1^ for CA33 to 124.79 µg mL^−1^ for CA34. Carbendazim values ranged from 40.69 µg mL^−1^ for *Colletotrichum* sp. CA15 to 218.92 µg mL^−1^ for CA34. Basic copper sulfate showed the widest numerical EC_50_ range, from 71.92 µg mL^−1^ for *Diaporthe* sp. CB5 to 699.68 µg mL^−1^ for CA3; the estimates for CA3 and CA17 exceeded the highest tested concentration of 600 µg mL^−1^ and were therefore model-derived extrapolations.

Bordeaux mixture showed consistently weak inhibition over the tested range of 1–200 µg mL^−1^. The maximum mean inhibition observed for Bordeaux mixture was 11.88%, and none of the seven isolates reached 50% inhibition; therefore, no EC_50_ was estimated for Bordeaux mixture (Figure 3; Table S2).

### 3.3. Antagonistic Screening Results and Broad-Spectrum Activity of CD103

The 45 mangrove-derived actinomycetes showed a broad range of antagonistic activity against *C. gloeosporioides* CCG3, with mean area-based inhibition ranging from 0 to 99.21% (Figure 4; Table S3). Twenty-two strains reached or exceeded the 50% screening reference. CD103 showed the highest mean inhibition (99.21 ± 0.40%) and was selected for subsequent breadth testing. Complete dual-culture plate phenotypes for all 45 candidate actinomycetes are shown in Figure S3.

CD103 was subsequently tested against 21 necrosis-inducing isolates spanning eight genera. Complete inhibition was observed for 19 of the 21 isolates (Figure 5). The two exceptions were *Fusarium* sp. CA32 and *Alternaria* sp. CA35, which retained limited residual growth and showed area-based inhibition of 85.04 ± 2.49% and 97.10 ± 0.80%, respectively. Thus, CD103 showed strong in vitro antagonistic activity against the tested isolates across all eight genera represented in the collection. All seven isolates evaluated in the fungicide sensitivity assays met the criterion for complete inhibition by CD103 in dual culture.

CD103 also met the CI criterion against all six reference plant pathogens, including *B. cinerea*, *F. oxysporum*, *C. gloeosporioides*, *C. musae*, *P. cinnamomi*, and *P. nicotianae* (Figure 6). The 1395 bp 16S rRNA gene sequence of CD103 showed 100% similarity to the type strain *Streptomyces angustmyceticus* NRRL B-2347^T^ in EzBioCloud and was deposited in GenBank under accession number PV420955.1. The isolate is referred to here as *Streptomyces* sp. CD103.

## 4. Discussion

*Colletotrichum* dominated the necrosis-inducing fungi recovered from coffee anthracnose-like lesions in Wanning, consistent with previous studies of coffee anthracnose in Hainan and elsewhere in China [1,2]. The recovery of seven additional genera also agrees with reports of *Fusarium*, *Diaporthe*, and other fungi from symptomatic coffee tissues [3]. This collection therefore provided a range of lesion-associated targets for antifungal testing. ITS comparisons with type-material reference sequences and phylogenetic analysis were used to place the isolates at genus level; species delimitation within closely related *Colletotrichum* complexes requires greater resolution than ITS alone generally provides [4]. The observed genus frequencies describe a culture-dependent collection from one site and one sampling date. Wound-induced necrosis provided a functional basis for selecting test isolates, but infection of unwounded leaves was not assessed. Their individual contributions to natural disease, and the mechanisms underlying the shared necrosis phenotype, remain unresolved.

Fungicide sensitivity varied substantially among the seven selected isolates, as reported for *Colletotrichum* associated with rubber [6], strawberry [7,8], persimmon [22], and soybean [23]. Thiophanate-methyl had the lowest EC_50_ for every isolate in the panel, although its values ranged from 0.57 to 22.53 µg mL^−1^. These low EC_50_ values contrast with reports of thiophanate-methyl resistance in strawberry-associated *Colletotrichum* [8], highlighting that fungicide sensitivity can vary markedly among isolates and study systems. Our panel comprised four *Colletotrichum* isolates and one each of *Lasiodiplodia*, *Clonostachys*, and *Diaporthe*, selected to retain genus-level diversity while including multiple *Colletotrichum* isolates with distinct ITS sequence profiles; all seven produced clear wound-induced necrosis under the assay conditions. Its mean EC_50_ values summarize these selected isolates, while the individual profiles identify differences that would be obscured by considering the means alone.

The relative sensitivity of an isolate also changed among fungicides. *Clonostachys* sp. CA34 had the highest EC_50_ values for carbendazim, thiophanate-methyl, and chlorothalonil, whereas *Lasiodiplodia* sp. CA33 was particularly sensitive to mancozeb and chlorothalonil but showed a much higher EC_50_ for basic copper sulfate. The responses to the two methyl benzimidazole carbamate (MBC) fungicides warrant consideration together because carbendazim and thiophanate-methyl share a β-tubulin-associated mode of action. Experimental reconstruction of the E198A substitution in *C. siamense* demonstrated its role in thiophanate-methyl resistance [24], and resistance to both compounds has been documented in *Fusicoccum amygdali* [25]. The relatively high EC_50_ values of CA34 for both compounds do not establish acquired resistance or a particular target-site mechanism. Their shared mode of action also limits the rationale for alternating these compounds in resistance management. Benzimidazole sensitivity has historically served as an auxiliary phenotypic character in *Colletotrichum* characterization, particularly in distinguishing isolates assigned to *C. acutatum* and *C. gloeosporioides* [26,27,28]. Cacciola et al. [29] also found low benomyl sensitivity in *C. ocimi* and *C. destructivum* isolates and the tested members of the *C. acutatum* complex, compared with high sensitivity in the tested *C. gloeosporioides* complex representatives. However, Talhinhas et al. [28] reported benomyl-sensitive isolates that grouped with *C. acutatum* in molecular analyses, demonstrating that this association is not absolute. Our genus-level assignments and the use of carbendazim and thiophanate-methyl rather than benomyl preclude a direct comparison with these diagnostic patterns.

The distinction between growth inhibition in culture and disease control is illustrated by Peres et al. [30]: benomyl only partially inhibited *C. acutatum* colony growth, yet applications before or shortly after inoculation substantially reduced citrus postbloom fruit drop. Their results show that the infection stage and timing of exposure can affect efficacy even when inhibition in culture is incomplete. Copper fungicides act primarily as surface protectants, with performance influenced by copper availability and deposition on plant surfaces [31]. The present assay measured established mycelial growth rather than spore germination, so the weak Bordeaux mixture response describes a specific developmental endpoint and does not resolve its protective efficacy on coffee plants. Differences from basic copper sulfate may reflect formulation properties, copper availability, and the different concentration ranges tested. The extrapolated EC_50_ values for CA3 and CA17 further limit precise comparisons at the upper end of the basic copper sulfate response range.

CD103 was selected for the highest mean inhibition of *C. gloeosporioides* CCG3 among the 45 actinomycetes, and its activity extended across the subsequent test panel. A total of 19 of the 21 necrosis-inducing isolates spanning eight genera met the complete-inhibition criterion, while *Fusarium* sp. CA32 and *Alternaria* sp. CA35 retained limited growth. All seven isolates used in the fungicide assays also met this criterion in dual culture with CD103, despite their differing fungicide sensitivity profiles. This overlap supports the breadth of target coverage; differences in exposure conditions and growth measurements preclude a direct comparison of potency between the chemical and biological treatments. Activity against the six reference plant pathogens further extended the range of susceptible targets. The mangrove-derived strain HSL-9B reduced postharvest mango anthracnose [12], while SCA3-4 produced extracts active against 13 phytopathogenic fungi [13]. Broad antifungal activity has also been reported for STR-1 [32], and B-1662 was evaluated against anthracnose and bitter rot in host assays [33]. Although the 1395 bp 16S rRNA gene sequence of CD103 was identical to the corresponding sequence of the type strain *Streptomyces angustmyceticus* NRRL B-2347^T^, this marker alone may not resolve closely related *Streptomyces* species [34]. We therefore retain the designation *Streptomyces* sp. CD103.

The active components responsible for CD103-mediated inhibition were not identified in this study. Antifungal metabolites have been reported from a mangrove-derived *Streptomyces hydrogenans* [35], extracellular enzymes and metabolites from *S. hygroscopicus* SRA14 [36], and antifungal volatile compounds from other *Streptomyces* strains [37]. These processes provide candidates for targeted investigation in CD103, potentially alongside competition within the shared culture medium. In the four-point confrontation assay, inhibition reflects the net interaction between the organisms under sustained antagonist exposure. Moreover, complete inhibition in most pairings leaves little scope for ranking the sensitivity of those targets under the same conditions. Cell-free assays and separation of diffusible and volatile effects would help resolve these responses. If activity is retained in cell-free preparations, bioassay-guided fractionation coupled with LC–MS profiling could link active fractions to candidate compounds.

Evaluating antifungal responses across this local collection extended the assessment beyond the predominant *Colletotrichum* isolates. The fungicide assays revealed contrasting sensitivity profiles, while CD103 retained strong in vitro activity across all eight genera tested. Progression from antagonism assays to host protection has been demonstrated for coffee anthracnose with *Trichoderma asperellum* GD040 [38] and for *Streptomyces*-based anthracnose control in other crops [33,39,40,41]. For CD103, the decisive next step is to establish whether this target coverage translates into reduced disease on coffee leaves and whole plants under realistic application conditions. Such evidence would provide a basis for formulation development and compatibility testing with fungicides having distinct modes of action.

## 5. Conclusions

Coffee anthracnose-like lesions sampled in Wanning yielded 39 necrosis-inducing fungal isolates spanning eight genera, with *Colletotrichum* remaining numerically dominant. Fungicide sensitivity among the seven selected isolates varied with both isolate identity and the compound tested, illustrating the limitation of using a single isolate to describe the response range observed in this panel. CD103 showed strong in vitro antagonistic activity against 21 necrosis-inducing isolates spanning all eight genera and against all six additional reference plant pathogens. These results support the use of diverse target panels when comparing fungicide sensitivity or assessing the breadth of a candidate antagonist. CD103 warrants further evaluation as a biocontrol candidate, with disease suppression on coffee leaves and whole plants as the next priority.

## Figures and Tables

**Figure 1:**
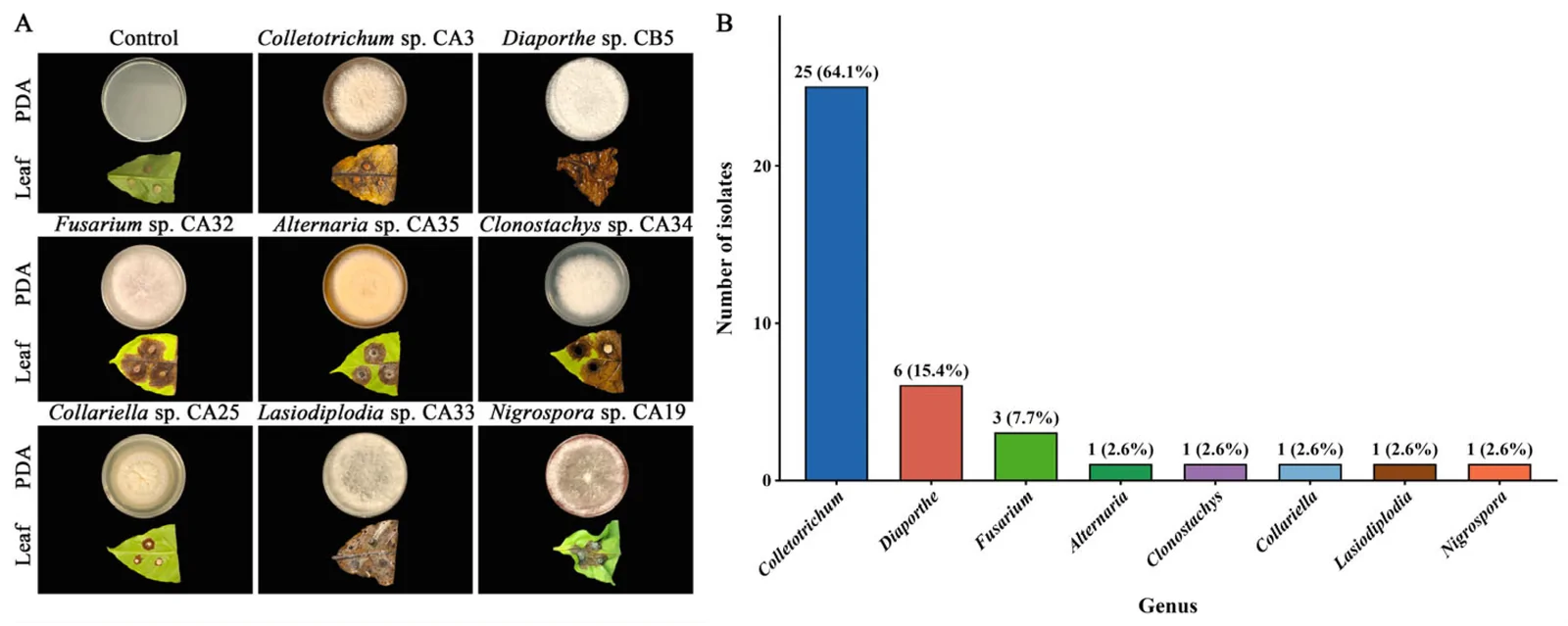
Representative colony morphologies, wound-induced necrosis phenotypes, and genus-level composition of necrosis-inducing fungi recovered from coffee anthracnose-like lesions. (**A**) Representative PDA colony morphologies and corresponding wound-induced necrosis phenotypes on detached coffee leaves for the eight genus-level groups. Representative isolates are *Colletotrichum* sp. CA3, *Diaporthe* sp. CB5, *Fusarium* sp. CA32, *Alternaria* sp. CA35, *Clonostachys* sp. CA34, *Collariella* sp. CA25, *Lasiodiplodia* sp. CA33, and *Nigrospora* sp. CA19. The control received a sterile PDA plug. Colony and leaf phenotypes are shown in the upper and lower rows of each group, respectively. (**B**) Genus-level distribution of the 39 necrosis-inducing isolates. Numbers above bars indicate isolate numbers, with percentages of the total in parentheses. Complete colony and wound-induced necrosis phenotypes for all 39 isolates are shown in Figure S1. PDA, potato dextrose agar.

**Figure 2:**
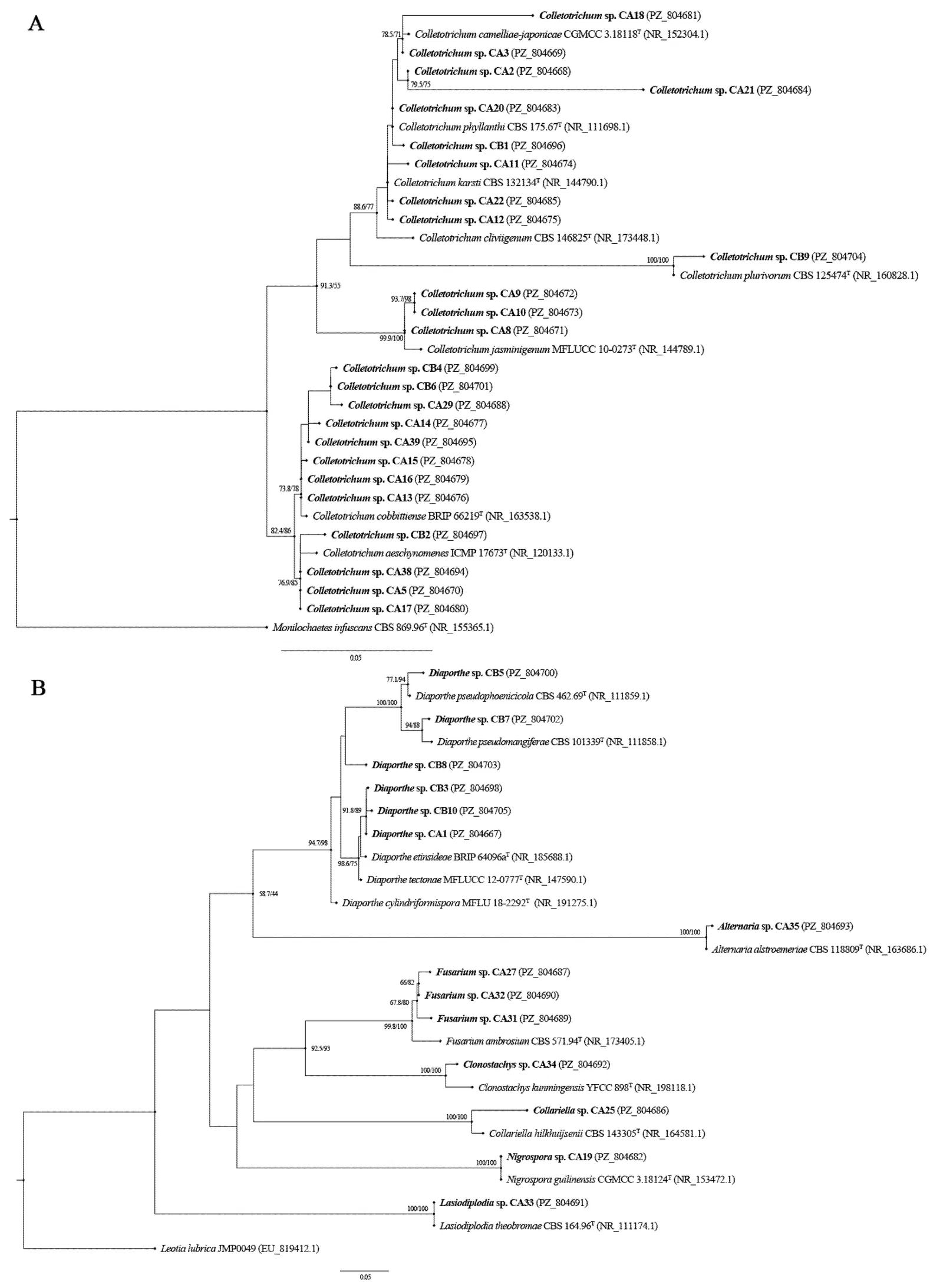
Maximum-likelihood phylogenies of fungi recovered from coffee anthracnose-like lesions based on ITS sequences. (**A**) Phylogenetic placement of the 25 *Colletotrichum* isolates. (**B**) Phylogenetic placement of the 14 isolates assigned to other genera. Study isolates are shown in bold and labeled with their genus-level assignment, isolate code, and GenBank accession number, with each isolate displayed as a separate tip. Reference sequences are shown with their taxonomic names, strain identifiers, and GenBank accession numbers. T denotes reference sequences derived from type material. Paired values adjacent to internal nodes indicate SH-aLRT/UFBoot support (%). *Monilochaetes infuscans* CBS 869.96 and *Leotia lubrica* JMP0049 were used as outgroups in panels (**A**) and (**B**), respectively. Scale bars indicate substitutions per site. ITS, internal transcribed spacer; SH-aLRT, SH-like approximate likelihood ratio test; UFBoot, ultrafast bootstrap.

**Figure 3:**
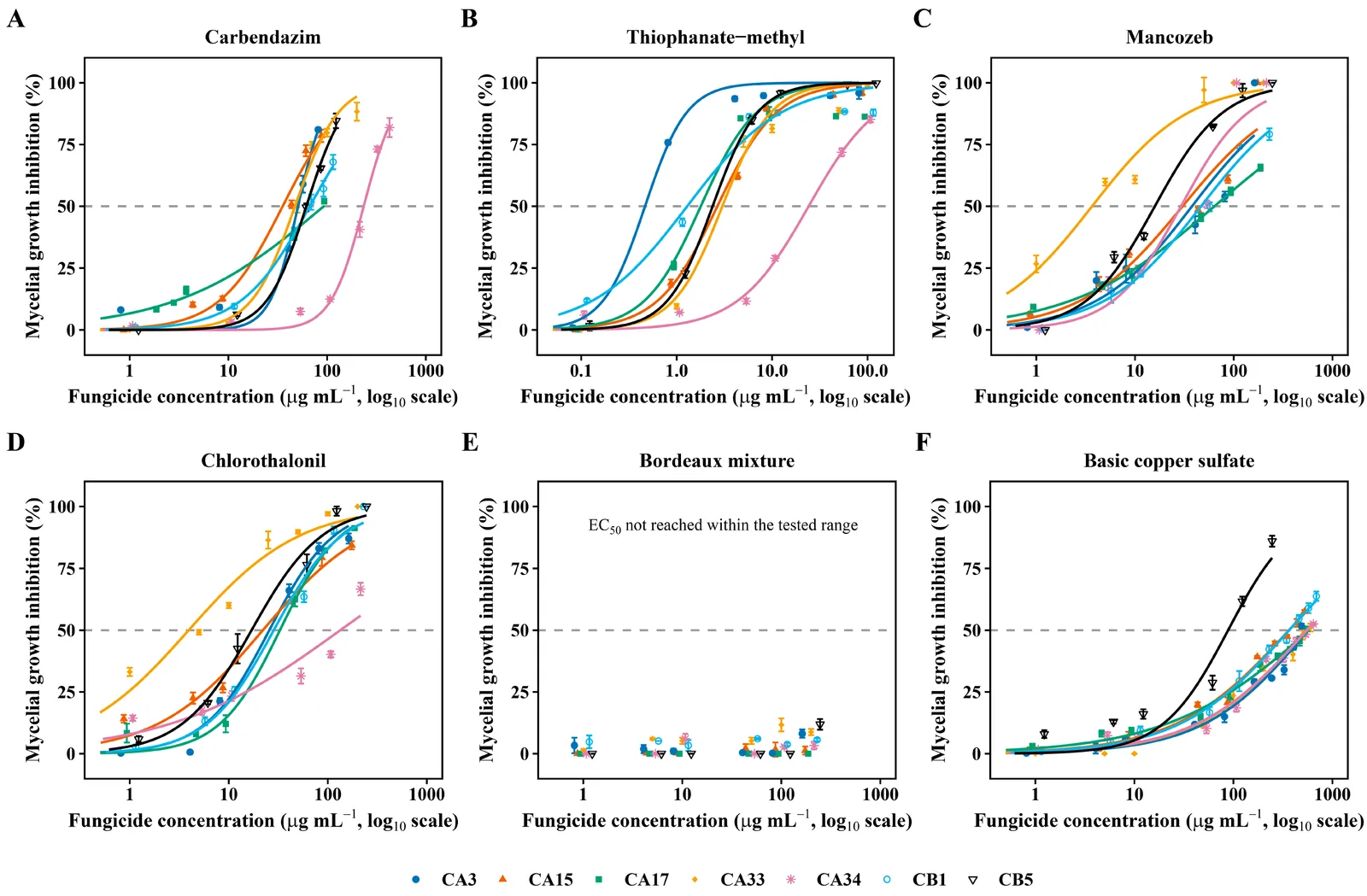
Concentration–response profiles of six fungicides against seven selected fungal isolates recovered from coffee anthracnose-like lesions. (**A**) Carbendazim; (**B**) thiophanate-methyl; (**C**) mancozeb; (**D**) chlorothalonil; (**E**) Bordeaux mixture; and (**F**) basic copper sulfate. Symbols and error bars represent mean mycelial growth inhibition ± SD from three independent plates at each tested concentration. For carbendazim, thiophanate-methyl, mancozeb, chlorothalonil, and basic copper sulfate, solid curves represent concentration–response relationships fitted using the nonlinear least-squares Hill model. Bordeaux mixture did not reach 50% inhibition within the tested concentration range; therefore, no EC_50_-based Hill model was fitted. The same color and symbol combination denotes the same fungal isolate across all panels. Horizontal dashed lines indicate 50% mycelial growth inhibition. Fungicide concentrations are plotted on a log_10_ scale. Representative plate phenotypes are shown in Figure S2. SD, standard deviation; EC_50_, concentration causing 50% inhibition of mycelial growth.

**Figure 4:**
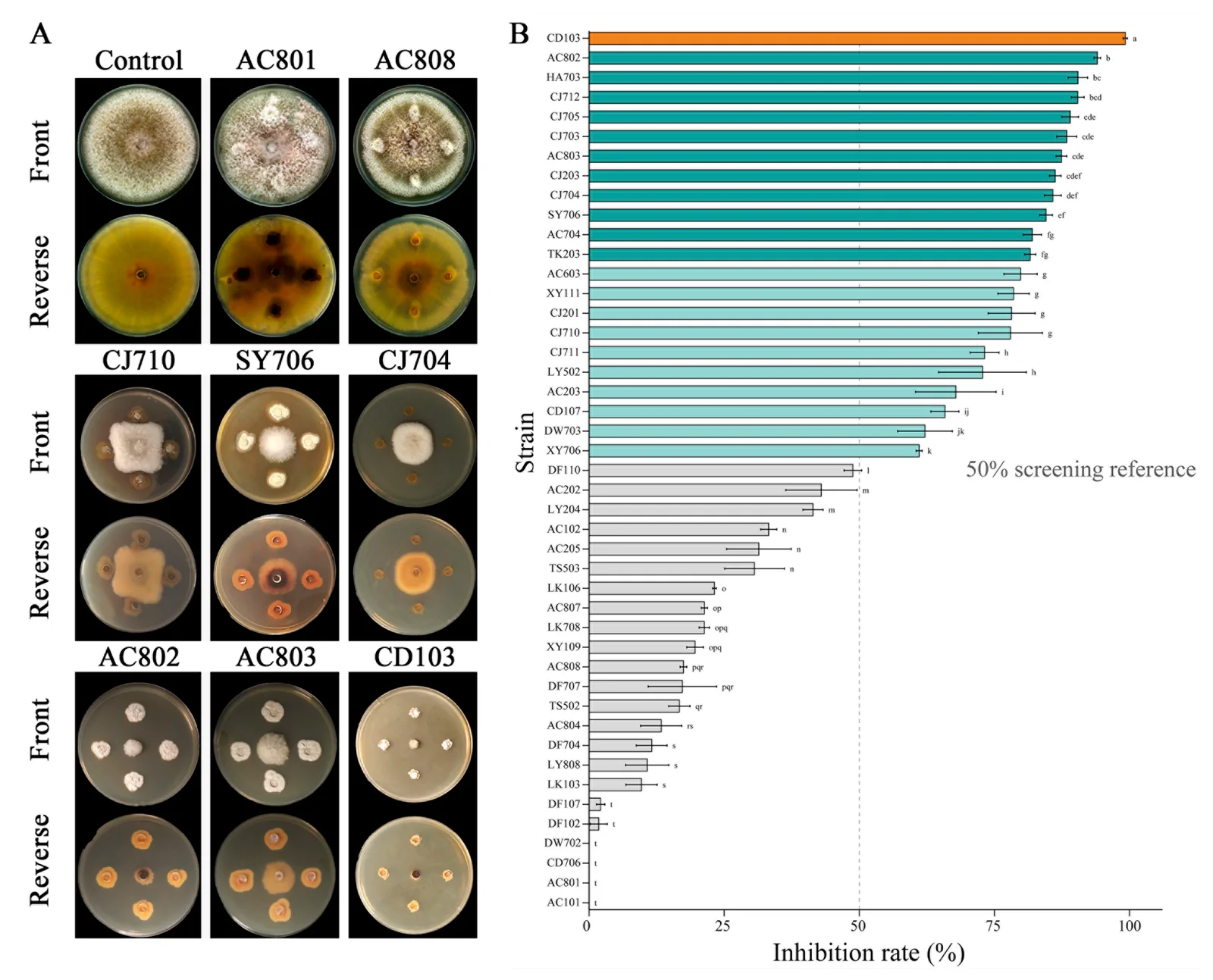
Dual-culture screening of 45 mangrove-derived actinomycetes against *Colletotrichum gloeosporioides* CCG3. (**A**) Representative dual-culture phenotypes from the actinomycete screening assay. The control shows CCG3 cultured without an actinomycete. For each candidate strain, four actinomycete agar blocks were positioned around the centrally inoculated CCG3 plug; the front and reverse sides of representative plates are shown. (**B**) Area-based inhibition rates of the 45 actinomycete strains against CCG3. Bars and error bars represent means ± SD (*n* = 3). CD103 is highlighted in orange; blue-green bars indicate mean inhibition ≥ 50%, whereas gray bars indicate mean inhibition < 50%. The vertical dashed line marks the 50% screening reference. Different lowercase letters indicate significant differences among strains according to Tukey’s HSD test (*p* < 0.05). Inhibition rates were calculated from fungal colony areas measured using ImageJ. SD, standard deviation; HSD, honestly significant difference.

**Figure 5:**
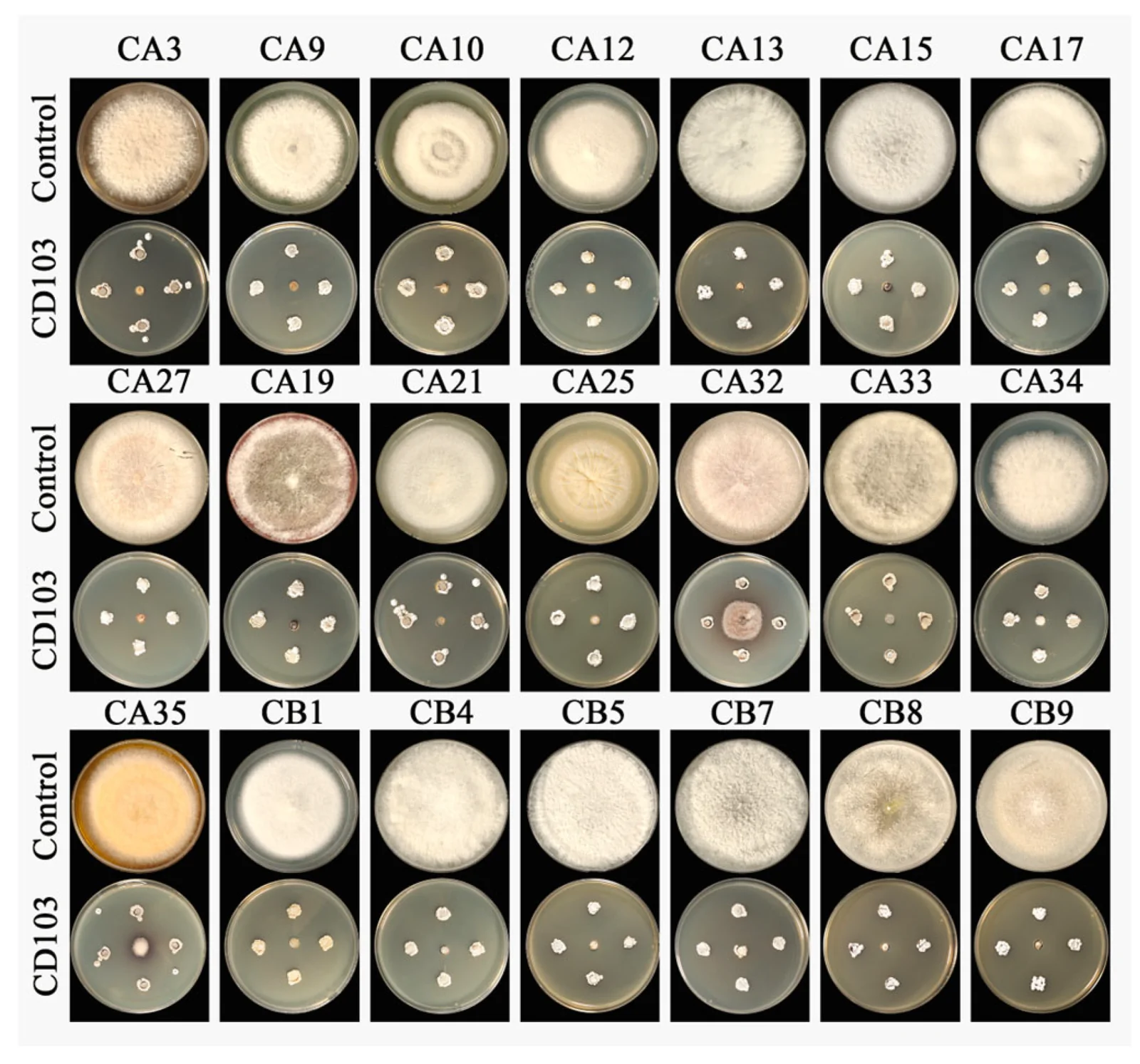
Dual-culture antagonism of *Streptomyces* sp. CD103 against 21 necrosis-inducing fungal isolates spanning eight genera, recovered from coffee anthracnose-like lesions. The panel comprised *Colletotrichum* spp. CA3, CA9, CA10, CA12, CA13, CA15, CA17, CA21, CB1, CB4, and CB9; *Diaporthe* spp. CB5, CB7, and CB8; *Fusarium* spp. CA27 and CA32; *Nigrospora* sp. CA19; *Collariella* sp. CA25; *Lasiodiplodia* sp. CA33; *Clonostachys* sp. CA34; and *Alternaria* sp. CA35. For each isolate, the upper plate shows the fungus-only control and the lower plate shows confrontation with CD103. *Fusarium* sp. CA32 and *Alternaria* sp. CA35 retained limited residual growth, with mean area-based inhibition of 85.04 ± 2.49% and 97.10 ± 0.80%, respectively; the remaining 19 isolates met the criterion for complete inhibition (CI). Each treatment comprised three independent plates, and representative plates are shown. CI was defined as no measurable fungal expansion beyond the initial inoculation block in any of the three plates.

**Figure 6:**
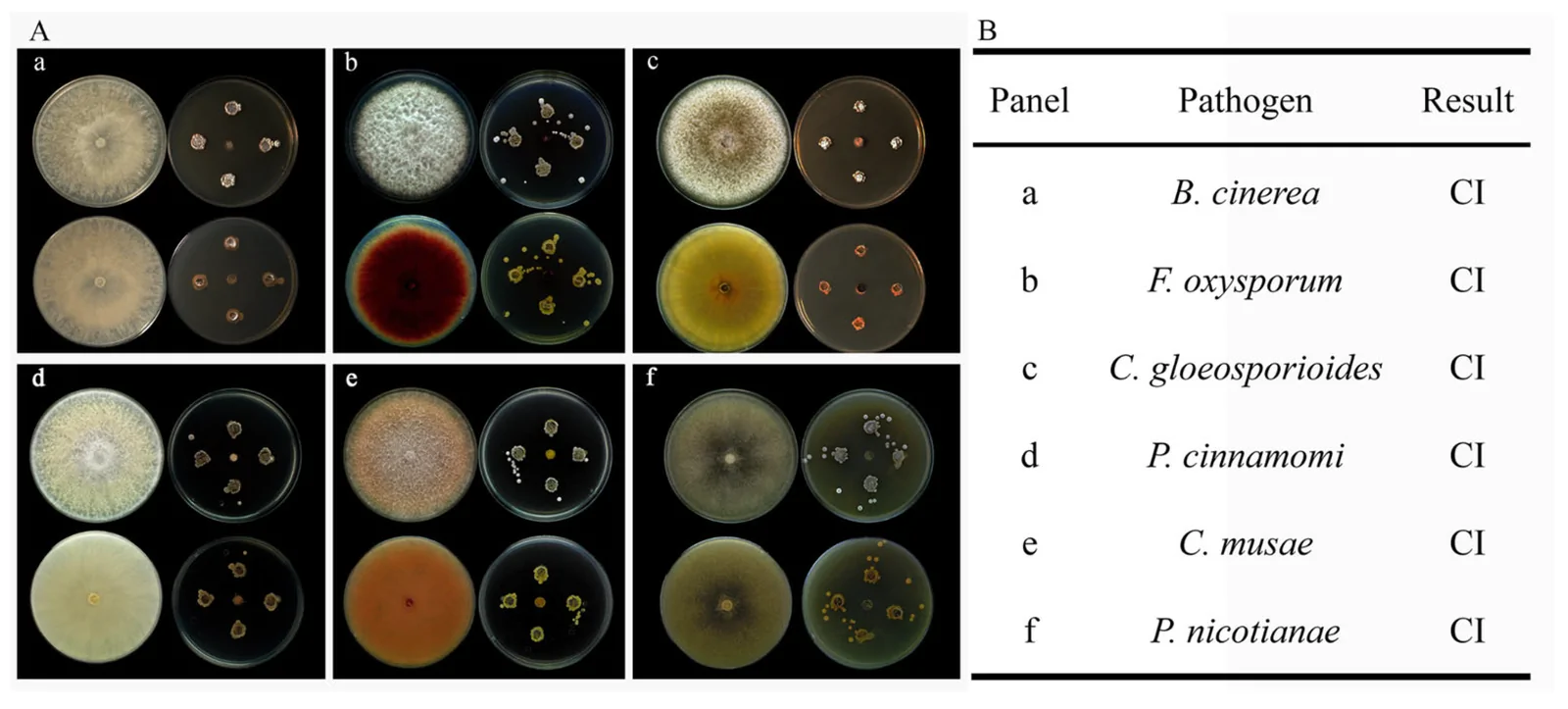
Antagonistic activity of *Streptomyces* sp. CD103 against six reference plant pathogens. (**A**) Representative dual-culture phenotypes. For each pathogen, the left plate shows the pathogen-only control and the right plate shows confrontation with CD103; the upper and lower rows show the front and reverse sides of the plates, respectively. Panels correspond to (**a**) *Botrytis cinerea*, (**b**) *Fusarium oxysporum*, (**c**) *Colletotrichum gloeosporioides*, (**d**) *Phytophthora cinnamomi*, (**e**) *Colletotrichum musae*, and (**f**) *Phytophthora nicotianae*. (**B**) Pathogen identities and corresponding inhibition phenotypes. CD103 met the criterion for complete inhibition (CI) against all six reference pathogens. CI was defined as no measurable pathogen expansion beyond the initial inoculation block in any of the three plates.

**Table 1:** Fungicides and concentration ranges used in the in vitro sensitivity assays.

Fungicide	Formulation	Mode of Action and FRAC Group	Tested Concentration Range (µg mL^−1^)
Carbendazim	50% WP	Single-site systemic MBC fungicide (FRAC 1)	1–400, isolate-dependent
Thiophanate-methyl	70% WP	Single-site systemic MBC fungicide (FRAC 1)	0.1–100
Mancozeb	80% WP	Organic multisite protectant (FRAC M03)	1–200
Chlorothalonil	75% WP	Organic multisite protectant (FRAC M05)	1–200
Bordeaux mixture	80% WP	Inorganic copper-based multisite protectant (FRAC M01)	1–200
Basic copper sulfate	27.12% SC	Inorganic copper-based multisite protectant (FRAC M01)	1–600, isolate-dependent

Note: Concentrations are expressed as nominal active-ingredient concentrations. Concentration series were selected on the basis of preliminary assays; exact concentrations tested for each isolate are provided in Table S2. WP, wettable powder; SC, suspension concentrate; MBC, methyl benzimidazole carbamate; FRAC, Fungicide Resistance Action Committee.

**Table 2:** Genus-level distribution of the 39 necrosis-inducing fungal isolates recovered from coffee anthracnose-like lesions and selection for dual-culture assays against CD103.

Genus-Level Group	Number of Isolates	Proportion (%)	Number of Isolates Tested Against CD103
*Colletotrichum*	25	64.1	11
*Diaporthe*	6	15.4	3
*Fusarium*	3	7.7	2
*Lasiodiplodia*	1	2.6	1
*Clonostachys*	1	2.6	1
*Alternaria*	1	2.6	1
*Nigrospora*	1	2.6	1
*Collariella*	1	2.6	1
Total	39	100	21

Note: Proportions were calculated relative to the 39 necrosis-inducing isolates. Percentages may not sum to exactly 100% because of rounding. The CD103 assay panel comprised 21 isolates selected based on genus-level assignments and preliminary ITS sequence comparisons, as described in Section 2.5.

**Table 3:** NLS-Hill-derived EC_50_ values of five fungicides against seven selected fungal isolates recovered from coffee anthracnose-like lesions.

Isolate	Genus-Level Assignment	Carbendazim EC_50_ (µg mL^−1^)	Thiophanate-Methyl EC_50_ (µg mL^−1^)	Mancozeb EC_50_ (µg mL^−1^)	Chlorothalonil EC_50_ (µg mL^−1^)	Basic Copper Sulfate EC_50_ (µg mL^−1^)
CA3	*Colletotrichum* sp.	62.48	0.57	46.50	31.58	699.68 *
CA15	*Colletotrichum* sp.	40.69	3.06	34.87	25.35	418.16
CA17	*Colletotrichum* sp.	99.01	1.92	68.29	35.18	633.85 *
CA33	*Lasiodiplodia* sp.	47.25	3.01	3.68	3.94	550.39
CA34	*Clonostachys* sp.	218.92	22.53	27.31	124.79	517.45
CB1	*Colletotrichum* sp.	58.17	1.12	40.67	24.77	318.60
CB5	*Diaporthe* sp.	50.05	1.93	12.92	13.81	71.92
Arithmetic mean		82.37	4.88	33.46	37.06	458.58

Note: Values are NLS-Hill-derived EC_50_ estimates expressed in µg mL^−1^. Arithmetic means are descriptive summaries across the seven selected isolates. Asterisks indicate model-derived EC_50_ estimates exceeding the highest tested concentration of 600 µg mL^−1^ for basic copper sulfate. Bordeaux mixture was excluded because inhibition did not reach 50% within the tested range of 1–200 µg mL^−1^ and no EC_50_ was estimated. NLS, nonlinear least squares; EC_50_, concentration causing 50% inhibition of mycelial growth.

## Data Availability

The summarized data supporting the findings of this study are provided in the article and its Supplementary Materials. The newly generated fungal ITS sequences have been deposited in GenBank under accession numbers PZ804667–PZ804705, and the 16S rRNA gene sequence of *Streptomyces* sp. CD103 is available under accession number PV420955.1.

## Supplementary Materials

The following supporting information can be downloaded at https://www.mdpi.com/article/10.3390/jof12100712/s1. Figure S1: Colony morphologies and wound-induced necrosis phenotypes of the 39 necrosis-inducing fungal isolates recovered from coffee anthracnose-like lesions. For each isolate, the upper image shows colony morphology on potato dextrose agar (PDA), and the lower image shows the corresponding phenotype on a wound-inoculated detached coffee leaf at 7 d after inoculation. The control received a sterile PDA plug and did not develop an expanding necrotic lesion. Isolate codes correspond to the genus-level assignments and ITS accession numbers listed in Table S1. Representative colony and leaf images are shown for each isolate. Figure S2: Concentration-dependent plate phenotypes of seven selected fungal isolates exposed to six fungicides. (A) Carbendazim; (B) thiophanate-methyl; (C) mancozeb; (D) chlorothalonil; (E) Bordeaux mixture; and (F) basic copper sulfate. The seven selected isolates were CA3, CA15, CA17, CA33, CA34, CB1, and CB5. Control plates contained fungicide-free PDA. Fungicide concentrations are indicated above the corresponding plates. Representative plates are shown for each isolate and concentration. Figure S3: Dual-culture plate phenotypes of all 45 mangrove-derived actinomycetes screened against *Colletotrichum gloeosporioides* CCG3. The CCG3-only plate served as the control. For each candidate actinomycete, four 5 mm × 5 mm actinomycete agar blocks were positioned equidistantly around the centrally inoculated CCG3 plug. The obverse and reverse sides of representative plates are shown for all 45 candidate strains. Strain codes are indicated above each plate pair. Table S1: Genus-level assignments, ITS accession numbers, and best-matching type-material ITS references of 39 necrosis-inducing fungal isolates recovered from coffee anthracnose-like lesions. Table S2: Concentration-specific mycelial growth inhibition of seven selected fungal isolates exposed to six fungicides, with final NLS-Hill model outputs where applicable. Table S3: Area-based inhibition of 45 mangrove-derived actinomycetes against *Colletotrichum gloeosporioides* CCG3, including mean ± SD inhibition values and Tukey HSD significance groups.

## Abbreviations

The following abbreviations are used in this manuscript:

ITS	Internal transcribed spacer
PDA	Potato dextrose agar
ISP2	International *Streptomyces* Project medium 2
SD	Standard deviation
EC_50_	Concentration causing 50% inhibition of mycelial growth
NLS	Nonlinear least squares
CI	Complete inhibition
FRAC	Fungicide Resistance Action Committee
MBC	Methyl benzimidazole carbamate
BIC	Bayesian information criterion
SH-aLRT	SH-like approximate likelihood ratio test
UFBoot	Ultrafast bootstrap
HSD	Honestly significant difference
