# Supplementary material for: Coffee Anthracnose-Associated Fungi from Wanning, Hainan: Fungicide Sensitivity and Broad-Spectrum Antagonism by Mangrove-Derived Streptomyces sp. CD103
Source: J Fungi (Basel). 2026 Sep 23;12(10):712. doi: 10.3390/jof12100712 (PMC13705787; doi:10.3390/jof12100712)
Supplement: Supplementary file 1 [file jof-12-00712-s001.zip › Table S1.pdf]

Table S1. Genus-level assignments, ITS accession numbers, and best-matching type-material ITS references of 39 necrosis-inducing fungal isolates recovered from coffee anthracnose-like lesions.

Isolate	Genus-level assignment	Study ITS accession	Best-matching type-material taxon	Reference strain/isolate	Reference accession	Sequence identity (%)
CA1	*Diaporthe* sp.	PZ804667	*Diaporthe etinsideae*	BRIP 64096a	NR_185688.1	98.76
CA2	*Colletotrichum* sp.	PZ804668	*Colletotrichum phyllanthi*	CBS 175.67	NR_111698.1	100.00
CA3	*Colletotrichum* sp.	PZ804669	*Colletotrichum cliviigenum*	CBS 146825	NR_173448.1	98.12
CA5	*Colletotrichum* sp.	PZ804670	*Colletotrichum cobbittiense*	BRIP 66219	NR_163538.1	99.13
CA8	*Colletotrichum* sp.	PZ804671	*Colletotrichum jasminigenum*	MFLUCC 10-0273	NR_144789.1	99.47
CA9	*Colletotrichum* sp.	PZ804672	*Colletotrichum jasminigenum*	MFLUCC 10-0273	NR_144789.1	99.47
CA10	*Colletotrichum* sp.	PZ804673	*Colletotrichum jasminigenum*	MFLUCC 10-0273	NR_144789.1	99.12
CA11	*Colletotrichum* sp.	PZ804674	*Colletotrichum cliviigenum*	CBS 146825	NR_173448.1	98.29
CA12	*Colletotrichum* sp.	PZ804675	*Colletotrichum phyllanthi*	CBS 175.67	NR_111698.1	100.00
CA13	*Colletotrichum* sp.	PZ804676	*Colletotrichum cobbittiense*	BRIP 66219	NR_163538.1	99.48
CA14	*Colletotrichum* sp.	PZ804677	*Colletotrichum cobbittiense*	BRIP 66219	NR_163538.1	99.31
CA15	*Colletotrichum* sp.	PZ804678	*Colletotrichum cobbittiense*	BRIP 66219	NR_163538.1	99.30
CA16	*Colletotrichum* sp.	PZ804679	*Colletotrichum cobbittiense*	BRIP 66219	NR_163538.1	99.47
CA17	*Colletotrichum* sp.	PZ804680	*Colletotrichum aeschynomenes*	ICMP 17673	NR_120133.1	99.65
CA18	*Colletotrichum* sp.	PZ804681	*Colletotrichum camelliae-japonicae*	CGMCC 3.18118	NR_152304.1	99.02
CA19	*Nigrospora* sp.	PZ804682	*Nigrospora guilinensis*	CGMCC 3.18124	NR_153472.1	100.00
CA20	*Colletotrichum* sp.	PZ804683	*Colletotrichum phyllanthi*	CBS 175.67	NR_111698.1	99.64
CA21	*Colletotrichum* sp.	PZ804684	*Colletotrichum karsti*	CBS 132134	NR_144790.1	96.83
CA22	*Colletotrichum* sp.	PZ804685	*Colletotrichum phyllanthi*	CBS 175.67	NR_111698.1	99.82
CA25	*Collariella* sp.	PZ804686	*Collariella hilkhuijsenii*	CBS 143305	NR_164581.1	97.51
CA27	*Fusarium* sp.	PZ804687	*Fusarium ambrosium*	CBS 571.94	NR_173405.1	97.73
CA29	*Colletotrichum* sp.	PZ804688	*Colletotrichum cobbittiense*	BRIP 66219	NR_163538.1	99.64
CA31	*Fusarium* sp.	PZ804689	*Fusarium ambrosium*	CBS 571.94	NR_173405.1	98.08
CA32	*Fusarium* sp.	PZ804690	*Fusarium ambrosium*	CBS 571.94	NR_173405.1	97.72
CA33	*Lasiodiplodia* sp.	PZ804691	*Lasiodiplodia theobromae*	CBS 164.96	NR_111174.1	100.00

Isolate	Genus-level assignment	Study ITS accession	Best-matching type-material taxon	Reference strain/isolate	Reference accession	Sequence identity (%)
CA34	*Clonostachys* sp.	PZ804692	*Clonostachys kunmingensis*	YFCC 898	NR_198118.1	98.25
CA35	*Alternaria* sp.	PZ804693	*Alternaria alstroemeriae*	CBS 118809	NR_163686.1	99.46
CA38	*Colletotrichum* sp.	PZ804694	*Colletotrichum aeschynomenes*	ICMP 17673	NR_120133.1	99.65
CA39	*Colletotrichum* sp.	PZ804695	*Colletotrichum cobbittiense*	BRIP 66219	NR_163538.1	99.30
CB1	*Colletotrichum* sp.	PZ804696	*Colletotrichum phyllanthi*	CBS 175.67	NR_111698.1	100.00
CB2	*Colletotrichum* sp.	PZ804697	*Colletotrichum aeschynomenes*	ICMP 17673	NR_120133.1	99.62
CB3	*Diaporthe* sp.	PZ804698	*Diaporthe etinsideae*	BRIP 64096a	NR_185688.1	98.76
CB4	*Colletotrichum* sp.	PZ804699	*Colletotrichum cobbittiense*	BRIP 66219	NR_163538.1	99.82
CB5	*Diaporthe* sp.	PZ804700	*Diaporthe pseudophoenicicola*	CBS 462.69	NR_111859.1	99.23
CB6	*Colletotrichum* sp.	PZ804701	*Colletotrichum cobbittiense*	BRIP 66219	NR_163538.1	99.30
CB7	*Diaporthe* sp.	PZ804702	*Diaporthe pseudomangiferae*	CBS 101339	NR_111858.1	98.19
CB8	*Diaporthe* sp.	PZ804703	*Diaporthe cylindriformispora*	MFLU 18-2292	NR_191275.1	97.13
CB9	*Colletotrichum* sp.	PZ804704	*Colletotrichum plurivorum*	CBS 125474	NR_160828.1	99.81
CB10	*Diaporthe* sp.	PZ804705	*Diaporthe tectonae*	MFLUCC 12-0777	NR_147590.1	99.07

Note: Study ITS accessions identify the newly generated sequences deposited in GenBank. Reference taxa, strain/isolate identifiers, accessions, and sequence identities refer to BLAST searches using the megablast option against the NCBI fungal ITS RefSeq database, restricted to sequences from type material. Reference taxon names indicate sequence matches and are not species-level assignments of the study isolates. ITS, internal transcribed spacer.
