# Supplementary material for: Coffee Anthracnose-Associated Fungi from Wanning, Hainan: Fungicide Sensitivity and Broad-Spectrum Antagonism by Mangrove-Derived Streptomyces sp. CD103
Source: J Fungi (Basel). 2026 Sep 23;12(10):712. doi: 10.3390/jof12100712 (PMC13705787; doi:10.3390/jof12100712)
Supplement: Supplementary file 1 [file jof-12-00712-s001.zip › Table S2.pdf]

Table S2. Concentration-specific mycelial growth inhibition of seven selected fungal isolates exposed to six fungicides, with final NLS-Hill model outputs where applicable.

Table S2A. Concentration-specific mycelial growth inhibition (%).

Fungicide	Concentration (μg mL⁻¹)	CA3	CA15	CA17	CA33	CA34	CB1	CB5
Carbendazim	1	8.12 ± 0.63	0.00 ± 0.00	—	0.00 ± 0.00	1.67 ± 0.36	1.04 ± 0.36	0.00 ± 0.00
Carbendazim	2	—	—	8.33 ± 0.95	—	—	—	—
Carbendazim	3	—	—	11.04 ± 0.95	—	—	—	—
Carbendazim	4	—	—	16.04 ± 1.57	—	—	—	—
Carbendazim	5	—	10.21 ± 0.95	—	—	—	—	—
Carbendazim	10	9.17 ± 0.95	12.71 ± 0.95	—	2.50 ± 0.63	3.33 ± 0.72	9.58 ± 0.95	6.25 ± 0.63
Carbendazim	40	—	—	—	—	—	39.17 ± 2.19	—
Carbendazim	50	32.92 ± 0.95	50.83 ± 1.57	37.50 ± 1.25	49.79 ± 2.37	7.50 ± 1.25	—	49.58 ± 1.57
Carbendazim	60	—	—	—	—	—	51.67 ± 3.08	—
Carbendazim	70	58.96 ± 3.44	72.50 ± 2.25	—	74.79 ± 1.30	—	—	65.42 ± 0.72
Carbendazim	80	—	—	—	—	—	57.08 ± 3.21	—
Carbendazim	100	81.04 ± 0.72	78.54 ± 2.53	52.08 ± 0.95	79.79 ± 1.57	12.50 ± 0.62	67.92 ± 2.95	84.58 ± 2.95
Carbendazim	200	—	—	—	88.33 ± 3.66	40.62 ± 3.12	—	—
Carbendazim	300	—	—	—	—	73.12 ± 1.08	—	—
Carbendazim	400	—	—	—	—	81.88 ± 3.90	—	—
Thiophanate-methyl	0.1	0.83 ± 0.95	0.00 ± 0.00	0.21 ± 0.36	0.00 ± 0.00	6.04 ± 1.57	11.88 ± 0.62	1.67 ± 1.91
Thiophanate-methyl	1	75.83 ± 0.95	18.96 ± 1.44	26.04 ± 1.57	9.58 ± 0.95	7.08 ± 0.36	43.54 ± 1.57	22.92 ± 1.91
Thiophanate-methyl	5	93.54 ± 0.95	62.29 ± 1.30	85.62 ± 0.62	76.25 ± 2.25	11.67 ± 0.95	86.25 ± 1.08	85.00 ± 1.65
Thiophanate-methyl	10	94.79 ± 0.95	89.58 ± 0.95	87.50 ± 2.72	81.46 ± 1.57	28.96 ± 0.95	87.92 ± 0.95	95.62 ± 1.25
Thiophanate-methyl	50	94.79 ± 0.95	95.21 ± 0.95	86.46 ± 0.36	88.75 ± 1.08	71.88 ± 1.65	88.33 ± 0.36	99.38 ± 0.62
Thiophanate-methyl	100	95.83 ± 2.37	95.83 ± 0.95	86.25 ± 0.62	98.75 ± 0.62	85.21 ± 1.30	87.92 ± 1.30	99.79 ± 0.36
Mancozeb	1	1.04 ± 0.72	6.46 ± 0.95	9.37 ± 0.63	26.67 ± 3.44	0.00 ± 0.00	0.83 ± 0.95	0.00 ± 0.00
Mancozeb	5	20.00 ± 3.48	18.54 ± 2.95	17.92 ± 1.44	59.79 ± 1.44	18.75 ± 2.72	15.83 ± 4.85	29.38 ± 2.17
Mancozeb	10	24.79 ± 5.67	31.04 ± 1.57	21.87 ± 2.86	60.83 ± 1.57	23.75 ± 2.25	22.92 ± 1.30	37.92 ± 1.30
Mancozeb	50	42.50 ± 3.48	48.54 ± 0.95	45.62 ± 1.65	97.08 ± 5.05	50.83 ± 1.57	49.79 ± 1.80	82.29 ± 0.36
Mancozeb	100	53.96 ± 2.01	61.04 ± 1.57	56.04 ± 1.57	100.00 ± 0.00	100.00 ± 0.00	74.17 ± 0.72	96.88 ± 2.72
Mancozeb	200	100.00 ± 0.00	100.00 ± 0.00	65.62 ± 1.25	100.00 ± 0.00	100.00 ± 0.00	79.17 ± 2.37	100.00 ± 0.00
Chlorothalonil	1	0.21 ± 0.36	14.17 ± 1.57	8.33 ± 3.82	33.12 ± 1.65	14.37 ± 1.08	2.71 ± 0.95	5.83 ± 1.30
Chlorothalonil	5	0.63 ± 0.62	22.50 ± 2.25	7.92 ± 0.72	49.17 ± 0.95	17.50 ± 1.87	13.12 ± 1.65	20.62 ± 0.63
Chlorothalonil	10	21.25 ± 1.25	26.67 ± 1.91	12.08 ± 3.44	60.00 ± 1.08	23.33 ± 2.01	25.83 ± 1.30	42.50 ± 5.96
Chlorothalonil	25	—	—	—	86.46 ± 3.44	—	—	—
Chlorothalonil	50	66.04 ± 2.60	62.08 ± 1.30	62.50 ± 2.86	89.79 ± 0.72	31.46 ± 3.08	63.54 ± 2.19	76.46 ± 4.25
Chlorothalonil	100	83.12 ± 2.25	79.38 ± 3.31	82.29 ± 0.95	97.08 ± 0.72	40.21 ± 1.30	90.42 ± 1.57	98.12 ± 1.88
Chlorothalonil	200	87.08 ± 2.01	84.38 ± 1.65	91.25 ± 0.62	100.00 ± 0.00	66.67 ± 2.53	100.00 ± 0.00	100.00 ± 0.00
Bordeaux mixture	1	3.33 ± 3.15	0.00 ± 0.00	0.00 ± 0.00	1.25 ± 0.62	0.00 ± 0.00	4.79 ± 2.60	0.00 ± 0.00

Fungicide	Concentration (μg mL⁻¹)	CA3	CA15	CA17	CA33	CA34	CB1	CB5
Bordeaux mixture	5	1.88 ± 1.65	0.21 ± 0.36	0.00 ± 0.00	6.04 ± 0.36	0.00 ± 0.00	5.13 ± 0.00	0.00 ± 0.00
Bordeaux mixture	10	1.04 ± 0.95	0.21 ± 0.36	0.00 ± 0.00	5.21 ± 1.30	6.04 ± 2.01	3.33 ± 2.19	0.00 ± 0.00
Bordeaux mixture	50	0.42 ± 0.36	2.08 ± 1.80	0.00 ± 0.00	5.21 ± 1.30	0.00 ± 0.00	6.04 ± 0.36	0.00 ± 0.00
Bordeaux mixture	100	0.21 ± 0.36	1.67 ± 2.89	0.00 ± 0.00	11.67 ± 2.60	2.71 ± 0.72	3.75 ± 0.63	0.00 ± 0.00
Bordeaux mixture	200	8.12 ± 1.65	1.25 ± 1.65	0.00 ± 0.00	8.75 ± 1.25	3.33 ± 1.57	5.62 ± 0.62	11.88 ± 2.17
Basic copper sulfate	1	0.21 ± 0.36	1.25 ± 1.25	3.12 ± 0.63	0.00 ± 0.00	1.67 ± 0.36	0.83 ± 0.36	7.92 ± 1.57
Basic copper sulfate	5	2.08 ± 2.01	2.08 ± 0.95	8.13 ± 1.25	0.00 ± 0.00	7.29 ± 1.44	4.79 ± 0.95	12.92 ± 0.72
Basic copper sulfate	10	5.83 ± 1.30	6.04 ± 2.82	9.58 ± 0.95	0.00 ± 0.00	6.25 ± 0.63	9.38 ± 1.65	16.04 ± 1.91
Basic copper sulfate	50	11.88 ± 0.62	19.79 ± 0.95	14.79 ± 1.30	11.46 ± 0.95	10.21 ± 2.01	16.67 ± 2.19	28.75 ± 2.86
Basic copper sulfate	100	15.00 ± 2.25	20.62 ± 2.17	22.92 ± 2.60	23.54 ± 2.53	18.54 ± 1.57	29.38 ± 4.10	61.67 ± 1.91
Basic copper sulfate	200	29.17 ± 0.95	39.17 ± 0.36	36.04 ± 2.53	34.58 ± 0.95	38.33 ± 0.95	42.29 ± 1.57	86.04 ± 2.19
Basic copper sulfate	300	30.62 ± 0.62	44.58 ± 0.95	39.38 ± 1.25	39.38 ± 0.62	38.75 ± 1.65	45.83 ± 0.95	—
Basic copper sulfate	400	33.96 ± 1.91	47.50 ± 0.62	42.29 ± 0.95	40.21 ± 2.53	45.42 ± 1.57	54.58 ± 1.57	—
Basic copper sulfate	500	43.12 ± 1.08	52.92 ± 1.57	45.62 ± 1.88	48.12 ± 1.88	48.33 ± 0.95	58.96 ± 2.01	—
Basic copper sulfate	600	51.67 ± 0.95	57.50 ± 0.62	50.62 ± 0.62	51.04 ± 1.57	52.50 ± 0.62	63.75 ± 1.88	—

Note: Values are presented as mean ± SD of mycelial growth inhibition (%). An em dash indicates that the corresponding concentration was not tested for that isolate.

Table S2B. Final NLS-Hill model outputs.

Fungicide	Isolate	Tested range ($\mu g\ mL^{-1}$)	EC_{50} ($\mu g\ mL^{-1}$)	Hill slope (h)	R^2	RMSE	Model status
Carbendazim	CA3	1–100	62.48	3.0588	0.9643	5.368	Fitted
Carbendazim	CA15	1–100	40.69	1.3604	0.9854	3.779	Fitted
Carbendazim	CA17	2–100	99.01	0.5815	0.9866	1.968	Fitted
Carbendazim	CA33	1–200	47.25	1.9635	0.9879	3.952	Fitted
Carbendazim	CA34	1–400	218.92	2.6050	0.9899	3.178	Fitted
Carbendazim	CB1	1–100	58.17	1.2465	0.9969	1.376	Fitted
Carbendazim	CB5	1–100	50.05	2.1624	0.9958	2.142	Fitted
Thiophanate-methyl	CA3	0.1–100	0.57	1.9774	0.9859	4.073	Fitted
Thiophanate-methyl	CA15	0.1–100	3.06	1.3917	0.9917	3.473	Fitted
Thiophanate-methyl	CA17	0.1–100	1.92	1.4759	0.9475	8.102	Fitted
Thiophanate-methyl	CA33	0.1–100	3.01	1.6980	0.9773	5.891	Fitted
Thiophanate-methyl	CA34	0.1–100	22.53	1.1714	0.9893	3.295	Fitted
Thiophanate-methyl	CB1	0.1–100	1.12	0.8767	0.9538	6.381	Fitted
Thiophanate-methyl	CB5	0.1–100	1.93	1.8336	0.9998	0.544	Fitted
Mancozeb	CA3	1–200	46.50	0.9006	0.8680	11.439	Fitted
Mancozeb	CA15	1–200	34.87	0.8400	0.9013	9.660	Fitted
Mancozeb	CA17	1–200	68.29	0.5983	0.9964	1.243	Fitted
Mancozeb	CA33	1–200	3.68	0.8838	0.9585	5.582	Fitted
Mancozeb	CA34	1–200	27.31	1.2679	0.9240	10.773	Fitted
Mancozeb	CB1	1–200	40.67	0.8908	0.9875	3.292	Fitted
Mancozeb	CB5	1–200	12.92	1.2548	0.9882	4.072	Fitted
Chlorothalonil	CA3	1–200	31.58	1.3333	0.9886	3.942	Fitted
Chlorothalonil	CA15	1–200	25.35	0.8137	0.9782	4.153	Fitted
Chlorothalonil	CA17	1–200	35.18	1.4185	0.9908	3.418	Fitted
Chlorothalonil	CA33	1–200	3.94	0.7810	0.9470	5.571	Fitted
Chlorothalonil	CA34	1–200	124.79	0.5014	0.8608	6.577	Fitted
Chlorothalonil	CB1	1–200	24.77	1.2717	0.9848	4.639	Fitted
Chlorothalonil	CB5	1–200	13.81	1.2279	0.9869	4.192	Fitted
Bordeaux mixture	CA3	1–200	—	—	—	—	Not estimated
Bordeaux mixture	CA15	1–200	—	—	—	—	Not estimated
Bordeaux mixture	CA17	1–200	—	—	—	—	Not estimated
Bordeaux mixture	CA33	1–200	—	—	—	—	Not estimated
Bordeaux mixture	CA34	1–200	—	—	—	—	Not estimated
Bordeaux mixture	CB1	1–200	—	—	—	—	Not estimated
Bordeaux mixture	CB5	1–200	—	—	—	—	Not estimated

Fungicide	Isolate	Tested range (μg mL^{-1})	EC_{50} (μg mL^{-1})	Hill slope (h)	R^2	RMSE	Model status
Basic copper sulfate	CA3	1–600	699.68*	0.8211	0.9738	2.744	Extrapolated
Basic copper sulfate	CA15	1–600	418.16	0.7552	0.9894	2.126	Fitted
Basic copper sulfate	CA17	1–600	633.85*	0.5954	0.9865	1.936	Extrapolated
Basic copper sulfate	CA33	1–600	550.39	0.7856	0.9841	2.458	Fitted
Basic copper sulfate	CA34	1–600	517.45	0.7733	0.9749	2.975	Fitted
Basic copper sulfate	CB1	1–600	318.60	0.7792	0.9940	1.736	Fitted
Basic copper sulfate	CB5	1–200	71.92	1.3119	0.9237	7.922	Fitted

Note: EC_{50} and Hill-slope parameters were estimated using bounded nonlinear least squares; R^2 and RMSE were calculated on the original inhibition scale. Asterisks indicate model-derived EC_{50} estimates above the highest tested concentration (600 μg mL^{-1}) for basic copper sulfate against CA3 and CA17. No NLS-Hill model was reported for Bordeaux mixture because inhibition did not reach 50% within 1–200 μg mL^{-1}.
