# Supplementary material for: Coffee Anthracnose-Associated Fungi from Wanning, Hainan: Fungicide Sensitivity and Broad-Spectrum Antagonism by Mangrove-Derived Streptomyces sp. CD103
Source: J Fungi (Basel). 2026 Sep 23;12(10):712. doi: 10.3390/jof12100712 (PMC13705787; doi:10.3390/jof12100712)
Supplement: Supplementary file 1 [file jof-12-00712-s001.zip › Table S3.pdf]

Table S3. Area-based inhibition of 45 mangrove-derived actinomycetes against *Colletotrichum gloeosporioides* CCG3.

Actinomycete strain	Area-based inhibition (%)	Tukey HSD group
CD103	99.21 ± 0.40	a
AC802	94.02 ± 0.63	b
HA703	90.45 ± 1.79	bc
CJ712	90.41 ± 1.17	bcd
CJ705	89.02 ± 1.54	cde
CJ703	88.37 ± 1.85	cde
AC803	87.39 ± 0.99	cde
CJ203	86.25 ± 1.07	cdef
CJ704	85.81 ± 1.54	def
SY706	84.57 ± 1.19	ef
AC704	82.00 ± 1.70	fg
TK203	81.62 ± 1.02	fg
AC603	79.83 ± 3.08	g
XY111	78.54 ± 2.89	g
CJ201	78.18 ± 4.34	g
CJ710	77.96 ± 5.94	g
CJ711	73.17 ± 2.66	h
LY502	72.79 ± 8.12	h
AC203	67.86 ± 7.46	i
CD107	65.84 ± 2.60	ij
DW703	62.15 ± 5.08	jk
XY706	61.08 ± 0.54	k
DF110	48.81 ± 1.62	l
AC202	42.98 ± 6.59	lm
LY204	41.44 ± 1.87	m
AC102	33.25 ± 1.50	n
AC205	31.41 ± 5.98	n
TS503	30.61 ± 5.53	n
LK106	23.21 ± 0.36	o
AC807	21.34 ± 0.57	op
LK708	21.31 ± 0.96	opq
XY109	19.61 ± 1.53	opq
AC808	17.47 ± 0.62	pqr
DF707	17.27 ± 6.34	pqr
TS502	16.71 ± 1.98	qr
AC804	13.34 ± 3.81	rs
DF704	11.58 ± 2.85	s
LY808	10.76 ± 4.00	s
LK103	9.70 ± 2.89	s
DF107	2.15 ± 0.78	t
DF102	1.79 ± 1.57	t
AC101	0.00 ± 0.00	t
AC801	0.00 ± 0.00	t
CD706	0.00 ± 0.00	t
DW702	0.00 ± 0.00	t

Note: Values are presented as mean ± SD of area-based inhibition (n = 3). Inhibition was calculated relative to the CCG3-only control, and negative values were set to 0. Different lowercase letters indicate significant differences among actinomycete strains according to one-way ANOVA followed by Tukey's HSD test ($p < 0.05$).
